# Duration of SHIV production by infected cells is not exponentially distributed: Implications for estimates of infection parameters and antiviral efficacy

**DOI:** 10.1038/srep42765

**Published:** 2017-02-16

**Authors:** Catherine A. A. Beauchemin, Tomoyuki Miura, Shingo Iwami

**Affiliations:** 1 Department of Physics, Ryerson University, Toronto, M5B 2K3, Canada; 2 Interdisciplinary Theoretical Science (iTHES) Research Group, RIKEN, Wako, 351-0198, Japan; 3 Institute for Virus Research, Kyoto University, Kyoto, 606-8507, Japan; 4 Department of Biology, Kyushu University, Fukuoka, 819-0395, Japan; 5 CREST and PRESTO, Japan Science and Technology Agency (JST), Saitama, 332-0012, Japan

## Abstract

The duration of the eclipse phase, from cell infection to the production and release of the first virion progeny, immediately followed by the virus-production phase, from the first to the last virion progeny, are important steps in a viral infection, by setting the pace of infection progression and modulating the response to antiviral therapy. Using a mathematical model (MM) and data for the infection of HSC-F cells with SHIV *in vitro*, we reconfirm our earlier finding that the eclipse phase duration follows a fat-tailed distribution, lasting 19 h (18–20 h). Most importantly, for the first time, we show that the virus-producing phase duration, which lasts 11 h (9.8–12 h), follows a normal-like distribution, and not an exponential distribution as is typically assumed. We explore the significance of this finding and its impact on analysis of plasma viral load decays in HIV patients under antiviral therapy. We find that incorrect assumptions about the eclipse and virus-producing phase distributions can lead to an overestimation of antiviral efficacy. Additionally, our predictions for the rate of plasma HIV decay under integrase inhibitor therapy offer an opportunity to confirm whether HIV production duration *in vivo* also follows a normal distribution, as demonstrated here for SHIV infections *in vitro*.

In this study, we sought to determine the distributions that describe the amount of time simian CD4^+^ T cells (HSC-F cells) spend in the eclipse and virus-producing phases, upon successful infection by the SHIV-KS661 strain of the simian-human immunodeficiency virus (SHIV). The eclipse phase is defined as the time which elapses between the entry of the first virion which successfully infects the cell and the release of the first virion produced by that newly infected cell. The virus-producing phase, which the cells enter immediately upon leaving the eclipse phase, is defined as the time which elapses between production of the first and last virions by an infected cell, often followed by apoptosis of that cell.

In previous work by our group^1^, we determined that the eclipse phase in this system is ~24 h in length. But most importantly, we determined that the time spent in the eclipse phase by SHIV-infected HSC-F cells was distributed according to a Gamma distribution with a shape parameter *n*_*E*_ ≈ 3.5, a finding we reconfirm herein using a different approach. This shape parameter, which we determine herein for both the eclipse and virus-producing phases, is important for a number of reasons. With respect to the eclipse phase, a shape parameter of *n*_*E*_ = 1 indicates that cells spend an exponentially-distributed amount of time in the eclipse phase, whereas 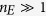 (e.g., *n*_*E*_ = 20) indicates they spend a normally-distributed time in that phase. An exponentially distributed eclipse length implies that some cells can theoretically produce virus immediately upon infection, which is not biologically possible^2^. On the other hand, assuming that the eclipse length is normally distributed amounts to assuming that all steps or successive processes involved in this phase — from successful virion entry into cells up until successful budding and release of the first virions — are of somewhat similar durations, and that said durations are short relative to the duration of the entire eclipse phase. Under these conditions, the eclipse phase, which corresponds to the total duration of all these processes, will follow a normal distribution, as per the central limit theorem.

Interestingly, in our previous investigation^1^, we found that, in fact, while this distribution for the eclipse phase is definitely not exponential (*n*_*E*_ ≠ 1), it is not normal either (*nE * 1). Instead, it lies in between (*n*_*E*_ = 4), i.e. a distribution that does enforce a delay before initiation of virus production, unlike the exponential distribution, but still keeps an exponential-like distribution for longer waiting times, unlike the very symmetric normal distribution. In other words, it allows for some cells to take a much longer than average time to produce their first virion progeny, referred to as a fat-tailed distribution. We believe this is because one of the processes involved in early SHIV replication is significantly longer than others, and possibly accounts for most of the eclipse phase’s duration. We suspect this step to be the integration of the reverse-transcribed SHIV DNA into the host cell’s genome. In contrast, we have shown that a virus like influenza A which replicates its segmented, negative-strand vRNA using its own polymerase, i.e. following a process devoid of this particular bottleneck, has an eclipse phase duration that follows a normal distribution^2^,^3^,^4^.

While we reconfirm this results for the eclipse phase distribution herein, our main focus in the present work is on determining, for the first time, the shape parameter which describes the phase immediately following the eclipse phase, namely the virus-producing phase, i.e., the duration for which cells infected with SHIV will produce and release virion progeny before this process is shut down, possibly as cells undergo apoptosis. An exponentially-distributed virus-producing phase (*n*_*I*_ = 1) would suggest that the process which leads to the termination of SHIV production *in vitro* is stochastic, completely independent of the time elapsed since cell infection. It would imply that cessation of virus production by SHIV-infected cells *in vitro* is *not* the result of accumulation of toxicity or exhaustion of intracellular host resources by virus replication, nor is it a virus production shut-down built into the virus replication cycle. It is biologically unlikely, and possibly unrealistic, that cessation of virus production not be the result of at least one of these processes. Yet, all mathematical models (MMs) of SHIV to date have invariably assumed the virus-producing phase, i.e. the duration of virus progeny production by SHIV-infected cells, to be exponentially distributed. Herein we show that, at least in SHIV-infected HSC-F cells*in vitro*, the duration of the virus-producing phase decisively does not follow an exponential distribution, but rather a normal-like distribution. We also evaluate the impact of assuming an exponentially vs a normally distributed virus-producing phase on the analysis of viral load decays observed in HIV patients treated with various antiviral regimens.

## Results

### Determining the distribution of the eclipse and virus-producing phases

**Figure 1:**
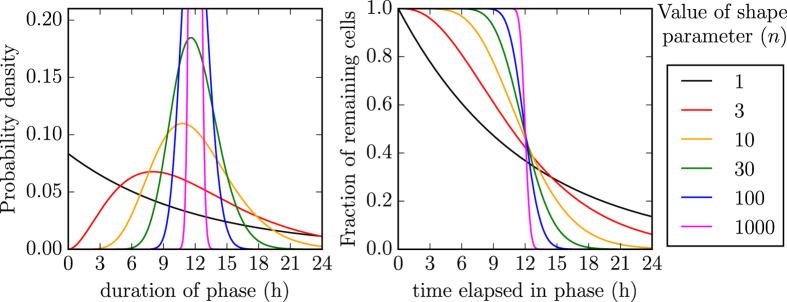
Effect of the Erlang distribution’s shape parameter. The Erlang distribution is used herein to describe the cell-to-cell variability in the time spent by SHIV-infected cells in the eclipse and virus-producing phases. As the Erlang shape parameter (*n*_*E*_ or *n*_*I*_ in MM (1), for the eclipse and virus-producing phases, respectively) is increased, the distribution of the phase duration shifts from an exponential (*n* = 1), to a fat-tailed (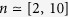), to a normal (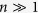), to a Dirac delta (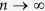) distribution. In these graphs, the mean time spent by cells in the phase (*τ*_*E*_ or *τ*_*I*_ in MM (1), respectively) is fixed (set to 12 h, chosen arbitrarily) as the shape parameter (*n*_*E*_ or *n*_*I*_) is varied. (Left) Probability density (*y*-axis) that a cell spend *x* hours in the (eclipse or infectious) phase. (Right) Fraction of cells (*y*-axis) which will remain in the phase for at least *x* hours.

Our goal is to determine the shape of the distributions describing the amount of time SHIV-infected cells spend in the eclipse phase, i.e. from successful virus entry to the release of the first virion, and then in the virus-producing phase, i.e. the duration of SHIV production from the release of the first to the last virion progeny by an infected cell. We use an Erlang distribution — a special case of the Gamma distribution in which the shape parameter must be an integer — to describe the cell-to-cell variability in the amount of time spent by SHIV-KS661 -infected cells in each of these two phases. By varying the value of the Erlang distribution’s shape parameter, one can shift the distribution from an exponential (=1) to a normal (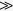
1) distribution, as illustrated in Fig. 1. As such, determination of the Erlang shape parameters for the eclipse and virus-producing phases will enable us to identify the shape of the distributions describing the time spent by SHIV-infected cells in these phases.

In previous work^1^, we used *in vitro* data from the infection of HSC-F cells infected with SHIV-KS661 at a concentration of 4.2 TCID_50_/cell to identify this shape parameter. Specifically, given the virus concentration in the inoculum, we assumed that all cells were simultaneously infected, and we used the cumulative fraction of cells that have entered the virus-producing phase — i.e., all cells that were either positive for the presence of the SHIV Nef protein or were no longer viable (have presumably died as a result of infection) — to identify this shape parameter for the duration of the eclipse phase. This non-dynamical approach is very attractive because the shape of the eclipse phase length distribution can be directly observed from this data alone, with the mathematical analysis providing a quantitative confirmation of what can already be seen.

Herein, we use a more indirect, dynamical approach by explicitly representing the kinetics of SHIV infection with MM (1), presented in the Methods section. This different approach enables us to relax the assumption that all cells were simultaneously infected by the initial inoculum, and allows us to also determine the distribution of the virus-producing phase duration, which until now has been assumed to be exponentially distributed. The experimental data used in the present analysis includes that used in our previous work^1^, as well as additional data collected as part of the previous experiment, but unused until now. Briefly, the experiment consisted in the infection of HSC-F cells with an inoculum containing SHIV-KS661 at concentrations of 4.2, 2.1, 1.1, 0.53, or 0.26 TCID_50_/cell. The total virus concentration (vRNA/mL), the fraction of viable HSC-F cells, and the fraction of virus-producing (i.e., SHIV Nef-positive cells) were determined at regular intervals over the course of the infection.

**Figure 2:**
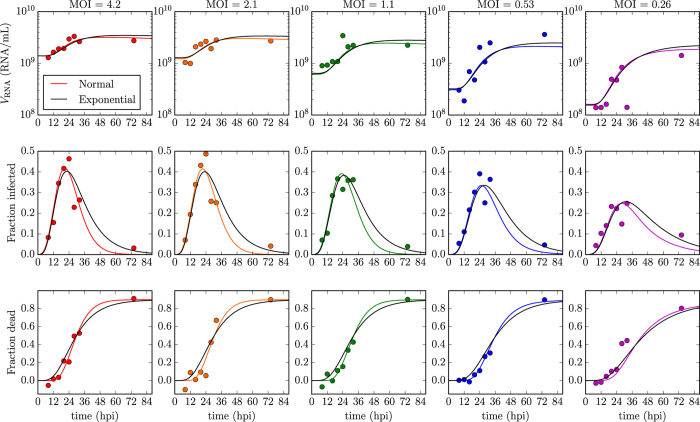
Time course of SHIV-KS661 infection of HSC-F cells and MM agreement. HSC-F cells were infected with SHIV-KS661 at a multiplicity of infection (MOI) or 4.2, 2.1, 1.1, 0.53, or 0.26 TCID_50_/cell, as indicated. At various times post-inoculation, the total viral load (top row), the fraction of virus-producing (Nef-positive) cells (middle row), and the fraction of dead cells (bottom rows) were determined as described in Methods. The lines correspond to the best-fits of MM (1) to this data set under the assumption that the virus-producing phase follows an exponential (*n*_*I*_ = 1, black line) or a normal-like (*n*_*I*_ = 20, coloured line) distribution (see Methods for MM parameter values). We find that a normal-like distribution for the duration of virus production yields a better agreement with the data (smaller SSR) than an exponential distribution.

The complete experimental data set is presented in Fig. 2, alongside simulated infection time courses from MM (1). The two solid lines in Fig. 2 correspond to the two best-fits of MM (1) to the data when we assume that the duration of the virus-producing phase is distributed either exponentially (*n*_*I*_ = 1), as is typically assumed in all existing MMs for (S)HIV, or that it is normally distributed (*n*_*I*_ = 20, chosen based on a more thorough best-fit analysis described below). While both distributions visually seem to provide reasonable fit to the data, the fit which assumes that SHIV-infected cells produce virus over a normally distributed amount of time is better (lower sum-of-squared residuals or SSR of 115, computed as per Eqn. (2)) than that assuming it is exponentially distributed (SSR of 135).

**Figure 3:**
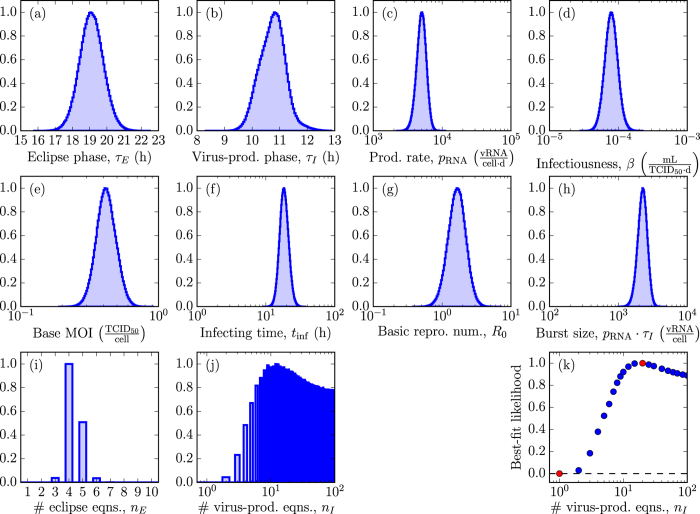
Posterior likelihood distributions of the key SHIV replication parameters. (**a**–**j**) A MCMC method was used to identify the posterior likelihood distributions (PLDs) for the value of MM (1)‘s parameters given the experimental data shown in Fig. 2. The likelihoods (*y*-axis of the PLDs) have been rescaled by the frequency of the mode. Note that the parameters corresponding to the number of eclipse (*n*_*E*_) and virus-producing (*n*_*I*_) equations can only take on integer values. Additionally, the PLD for parameter *n*_*I*_ in panel (j) is not a true PLD because *n*_*I*_ was constrained to be ∈[1, 100], wherein the upper-limit of 100 was chosen arbitrarily given that values for 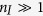 are statistically equivalently likely. (**k**) To supplement the MCMC analysis, a best-fit likelihood (BFL) was obtained via a series of nonlinear fits performed while holding parameter *n*_*I*_ fixed to values ranging from *n*_*I*_ = 1 to 100. The red dots indicate the best-fits for the exponential (*n*_*I*_ = 1) and normal-like (*n*_*I*_ = 20) distributed virus-producing phase shown in Fig. 2. The BFL was rescaled so that the maximum likelihood equals one (see Methods).

To investigate this further, we used a Markov chain Monte Carlo (MCMC) approach to determine the posterior likelihood distribution (PLD) for each of the MM parameters. In particular, we sought to determine the likelihood that the virus-producing phase is exponentially (*n*_*I*_ = 1) versus normally (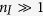) distributed, given the set of observed experimental data. These results are presented graphically in Fig. 3, and quantitatively in Table 1. We find that all parameters are robustly extracted (see Supplementary Fig. S1 for 2-parameter PLDs), with little to no correlations resulting in narrow PLDs. The MCMC analysis determined that newly infected HSC-F cells spent 19 h (95% credible region or CR of *τ*_*E*_ = 18–20 h) in the eclipse phase before they begin producing SHIV-KS661 progeny. Once underway, virus production proceeds at a rate of 5,000 (95% CR: *p*_RNA_ = 4,000–6,300) SHIV virion per cell per day which infected cells maintain for 11 h (95% CR: *τ*_*I*_ = 9.8–12 h) before presumably undergoing apoptosis. This results in a total burst size of 2,500 (95% CR: *p*_RNA_ · *τ*_*I*_ = 1,600–3,200) SHIV-KS661 virion produced by each SHIV-infected HSC-F cell over its virus-producing lifespan. Despite this high virus yield, infection progresses slowly with 18 h (95% CR: *t*_inf_ = 14–25 h) elapsed between the start of virion progeny production and infection of the first cell by that progeny, resulting in a modest basic reproductive number of 1.7 (95% CR: *R*_0_ = 0.91–2.9). We also confirm our earlier finding that the duration of the eclipse phase follows a fat-tailed distribution that is somewhere between exponential and normal, with a shape parameter value of 4 (95% CR: *n*_*E*_ = [3–5]), in agreement with the value of 3.5 obtained previously using a different approach^1^.

Of greatest interest, however, is the PLD of the parameter characterizing the shape of the distribution for the duration of the virus-production phase, *n*_*I*_. Because values of 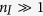 are statistically equivalently likely, we constrained our MCMC process to values of *n*_*I*_ ∈ [1, 100]. As such, the PLD for *n*_*I*_ is not a true posterior likelihood density. Nonetheless, this enables us to identify that the mode of the PLD is 12, with a 95% CR of [6, 97]. Furthermore, out of the >7,000,000 MCMC-accepted parameters, corresponding to roughly 100,000 independent parameter estimates (given our autocorrelation length of ~70), not a single one had *n*_*I*_ = 1. Therefore, based on Feldman and Cousins^5^, we can state with 95% confidence that the likelihood of an exponentially-distributed virus-producing phase (*n*_*I*_ = 1) is no more than 3 in 100,000 or <10^−4^.

In Fig. 3(k), we supplement the MCMC analysis by performing a nonlinear regression to the data presented in Fig. 2, while holding *n*_*I*_ fixed to values ∈ [1, 100]. Leaving parameter *n*_*I*_ as a free parameter to be fitted was not appropriate given that values of 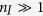 are all equivalently likely (causing the fitter to diverge) and the fact that *n*_*I*_ can only take on integer values (which causes the fitter to misbehave). We find that fits for *n*_*I*_ ∈ [9, 100] have a best-fit likelihood (BFL) >85% that of the very best BFL, obtained here for *n*_*I*_ = 20. In particular, we find the BFL increases monotonically from *n*_*I*_ = 1 to *n*_*I*_ = 20, with the BFL for *n*_*I*_ = 1 being <10^−4^ that for *n*_*I*_ = 20. In other words, both the PLD and BFL for parameter *n*_*I*_ unambiguously, statistically significantly exclude the possibility that SHIV-infected cells could produce virus progeny for an exponentially distributed (*n*_*I*_ = 1) amount of time. Instead, the duration of the virus-producing phase is consistent with a mildly fat-tailed or more normal-like distribution.

### Impact of the eclipse and virus-producing phase distributions on predicted viral load decays under antiviral therapy

The shape of the distribution for the time spent by cells in the eclipse and virus-producing phases can appear to be a theoretical concern of little relevance to our understanding of, and therapeutic approach to, HIV. However, the increasing prevalence of MM analyses, and the ability they confer to determine antiviral efficacy based solely on early viral load decays observed in treated patients, has made them an essential part of the toolkit used in developing, evaluating, and optimizing novel HIV therapies. All MMs must invariably make assumptions and simplifications which, occasionally, can lead to misinterpretations or inaccurate quantification. Here, we tackle one such simplification in the context of our findings regarding the shape parameters of the eclipse and virus-producing phase distributions.

To date, almost all MMs applied to the analysis of HIV infection kinetics have assumed that the durations of the eclipse phase and of virus production by HIV-infected cell are exponentially distributed. In other words, these MMs assume a cell that has just been infected can immediately begin producing virus, and that a cell which has just begun to produce and release HIV progeny is as likely to cease production and undergo apoptosis as one that has been producing virus for hours or even days. In the previous section, we established *in vitro* that the duration of the SHIV eclipse phase is fat-tailed distributed, and there is little reason to believe that this would not be the case *in vivo*. Though we have also established the duration of the virus-producing phase is normally distributed *in vitro*, this might not be the case *in vivo* where host factors and immune responses could abrogate or otherwise significantly affect the actual duration of virus production by HIV-infected cells. Therefore, it is important to understand how the decays predicted using the traditional MM (Exp,Exp), differ from those of the more biologically correct MMs with a fat-tailed distributed eclipse phase and either an exponential (Fat,Exp) or normally (Fat,Norm) distributed eclipse phase. Herein, we explore the impact of this finding on interpretations of antiviral efficacy based on observed patterns of early plasma viral load decay upon therapy initiation in HIV patients.

**Figure 4:**
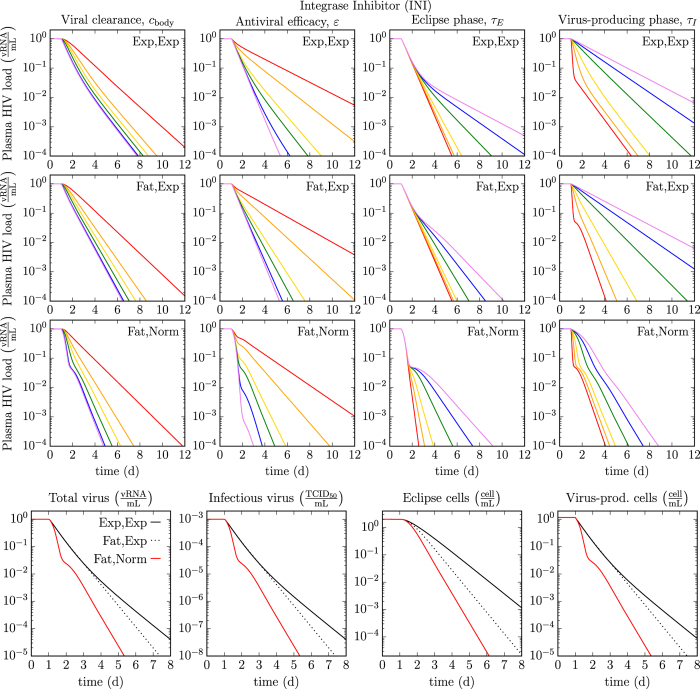
Comparison of viral load decays under integrase inhibitor therapy for different eclipse and virus-producing phase distributions. Using MM (3), we simulated the expected decays in plasma HIV viral load under therapy with an integrase inhibitor. The top three rows explore how four key *in vivo* viral kinetic parameters — rate of HIV viral clearance, antiviral efficacy, and the durations of the eclipse and virus-producing phases — affect the MM-predicted plasma HIV viral load decay under therapy (red to purple = lowest to highest parameter value), when assuming exponentially distributed eclipse and virus-producing phases (Exp, Exp, *n*_*E*_, *n*_*I*_ = [1, 1]), or a fat-tailed distributed eclipse phase with an exponential (Fat, Exp, *n*_*E*_, *n*_*I*_ = [4, 1]) or normally distributed (Fat, Norm, *n*_*E*_, *n*_*I*_ = [4, 12]) virus-producing phase. Values of the viral clearance rate (*c*_body_) of 1, 2, 3, 5, 20, and 100 h^−1^, of the antiviral efficacy (*ε*_IN_) of 0.5, 0.7, 0.9, 0.95, 0.99, and 0.999, of the eclipse (*τ*_*E*_) or virus-producing (*τ*_*I*_) phases of 1, 6, 12, 24, 36, and 48 h are explored. The bottom row explores the effects of the MM choice on the expected decay of plasma HIV total and infectious virus load, and eclipse and virus-producing cells (*c*_body_ = 23 h^−1^, *ε*_IN_ = 0.97, *τ*_*E*_ = 19 h, *τ*_*I*_ = 11 h). The simulated therapy is applied at day one and no pharmacokinetic delay has been added.

In Fig. 4, we compare the rate of decay of plasma HIV viral loads decays under antiviral therapy with an integrase inhibitors (INIs) such as raltegravir, as predicted by MMs. We explore how the MM-predicted HIV decay rates are impacted by four key infection kinetic parameters: the *in vivo* HIV clearance rate, antiviral efficacy, and the durations of the eclipse and virus-producing phases. We investigate how that impact in turn depends on the assumptions made regarding the distributions of the eclipse and virus-producing phases (Exp,Exp or Fat,Exp or Fat,Norm). The last row of Fig. 4 shows how, for given parameter values, the MM-predicted decay rates under INI therapy differ between these three assumptions. For example, the predicted total vRNA viral load under INI therapy differs by one to two orders of magnitude (10- to 100-fold) between the MMs with an Exp vs Normal virus-producing lifespan. We expect such a difference would be experimentally measurable.

The traditional (Exp,Exp) MM and the two variants considered here all predict a biphasic decay under monotherapy with a INI for most parameter values, as is typically observed in INI-treated HIV patients, without the need to resort to assuming one short-lived and one long-lived infected cell population^6^. For a given eclipse phase duration, the traditional MM predicts a lesser rate of viral load decay under therapy. As such, the traditional MM would likely underestimate the true eclipse phase duration. More generally, we find that having observed a given decay in a patient, use of the traditional MM (Exp,Exp) to analyse said decay will overestimate the true antiviral efficacy and/or the HIV clearance rate, and/or it will underestimate the true duration of the eclipse or virus-producing phase. The over/under-estimation is even greater if one further assumes that the virus-producing duration is normally (Fat,Norm) rather than exponentially (Fat,Exp) distributed. For example, the decay predicted under INI therapy at 90% efficacy by the (Fat,Norm) MM (yellow line) is predicted at an antiviral efficacy of ~99% by the traditional (Exp,Exp) MM (blue line). The 10-fold difference (10% vs 1% antiviral escape) in the estimates between these two different MMs would likely be statistically significant and could impact other MM predictions such as time to antiviral resistance emergence.

The MM which assumes a normally distributed duration of virus production (Fat,Norm) predicts a very sharp decay at a rate proportional to the viral clearance rate, which stops in a shoulder whose depth depends on the antiviral efficacy, followed by a second phase of decay whose rate depends on the duration of the eclipse phase. This shape is observed provided the viral clearance rate (*c*_body_) is greater than ~5 h^−1^ and the duration of virus production is less than ~11 h. While it is now believed the viral clearance rate is greater than 5 h^−1^, and likely as high as 23 h^−1 ^7, it is not clear whether or not the duration of virus production by HIV-infected cell *in vivo* is indeed less than 11 h. If it is not, the MM (Fat,Norm) predicts the duration of virus production would affect both the first and second phase of viral load decay, but not the depth of the shoulder.

## Discussion

Herein we have made use of a mathematical model (MM) combined with a Markov chain Monte Carlo approach to determine the shape of the distribution describing the time spent by SHIV-infected HSC-F cells in the eclipse and virus-producing phases. In previous work^1^, we determined that a Gamma shape parameter of 3.5 best describes the distribution of time spent by cells in the eclipse phase, i.e. the time elapsed between cell infection and the release of the first SHIV progeny by that cell. Herein, we reconfirm this finding (we found a value of 4 with a 95% credible region (CR) of [3–5]) using a more extensive data set and a different approach.

However, the primary aim of the present work was to extract the shape parameter for the phase which follows the eclipse phase, namely the virus-producing phase, i.e. the time elapsed between production of the first and last virus progeny by a productively SHIV-infected cell. MMs for (S)HIV infection kinetics have, up until this point, almost always assumed that the virus-producing phase is exponentially distributed. Herein, our experimental data, MM, and analysis, enable us to statistically exclude (probability < 10^−4^) the hypothesis that the duration of virus production by SHIV-infected HSC-F cells follows an exponential distribution. Instead, we find that the Gamma shape parameter for the distribution of time SHIV-cells spend in the virus-producing phase is ~12, consistent with a mildly fat-tailed or normal-like distribution (*n*_*I*_ = [6, 97], 95% CR). To our knowledge, this is the first time the virus-producing lifespan of SHIV-infected cells *in vitro* has ever been shown to follow a normal-like distribution, rather than an exponential distribution as has been assumed in all MMs for (S)HIV until now. This finding is consistent with earlier work by Petravic *et al*.^8^ which also suggests that the eclipse phase duration and virus-producing lifespan of HIV-infected cells *in vitro* are inconsistent with an exponential distribution. Due to the nature of the data used in their analysis, Petravic *et al*. assumed, rather than identified, that the duration of both the eclipse and virus-production phases followed a fat-tailed, log-normal-like distribution. Such a distribution is consistent with that identified here for the duration of the eclipse phase, but differs from the more normal-like distribution favoured herein by our MCMC analysis for the virus-production phase. However, since Petravic *et al*. assumed rather than determined the distribution, it is likely that a normally distributed virus-production phase would also successfully reproduce their data.

Use of a normally rather than an exponentially distributed virus-producing cell lifespan can also impact estimates of the key SHIV kinetic parameters from MM analysis. Our results establish that SHIV-infected cells begin progeny virus production and release approximately 19 h (*τ*_*E*_ ∈ [18, 20] h) post-infection, and cease approximately 11 h (*τ*_*I*_ ∈ [9.8, 12] h) after they have begun, or 30 h (*τ*_*E*_ + *τ*_*I*_ ∈ [28, 31] h) after the successful entry of the virion which infected them. This appears to be consistent with the distributions parametrised by Petravic *et al*.^8^ for the duration of the eclipse and virus-production phases of HIV-infected cells *in vitro* whose mode we visually estimate to be about 28 h (*τ*_*E*_) and 12 h (*τ*_*I*_), respectively. In contrast, our estimates for the duration of the virus-producing cell lifespan (*τ*_*I*_) obtained herein is 1.7–2.1× longer than that obtained in previous work^9^ wherein an exponentially-distributed virus-producing cell lifespan was assumed. Our estimate of the basic reproductive number, namely the number of secondary cells infected by the virus progeny produced by a single SHIV-infected cells over its virus-producing lifespan *in vitro*, was 1.7 (*R*_0_ ∈ [0.91, 2.9]), lower than previously estimated *in vivo* ranges of 4.3–13 assuming no eclipse phase or 5.4–54 assuming an exponential eclipse phase, both estimated in ref. 10, or 4–11 assuming no eclipse phase in ref. 11 for HIV-1 infections in human patients, and 2.2–4.6 assuming no eclipse phase in macaques infected with simian immunodeficiency virus (SIV)^12^.

MMs are widely used to analyse and interpret the decay of plasma HIV load in patients treated with antivirals. Hence, we also investigated the impact of assuming the eclipse and virus-producing phases are exponentially distributed, as is typically done, in analysing and interpreting said decays. Assuming the eclipse phase duration is exponentially-distributed, which is inconsistent with our findings herein, can cause an overestimation of the antiviral efficacy or an underestimation of the duration of the eclipse or virus-producing phases. The over/under-estimation is even more significant if the true duration of virus-production is normally, rather than exponentially distributed. Having found a 10-fold difference in the antiviral efficacy estimated by MMs with an exponential vs normally distributed virus-producing cell lifespan, we expect these parameter over/under-estimation from viral decay data observed under INI therapy would be statistically significant. In turn, these statistically significant differences in parameter value estimates could potentially have an impact on therapeutic decisions. Actual analysis of viral load decays under therapy using these different MMs is needed to confirm this possibility, and to identify which parameter(s) would be most significantly affected. Previous works analysing plasma viral load decays under antiviral therapy *in vivo*, such as that by Althaus *et al*. for HIV^13^ and by Rong *et al*. for hepatitis C virus^14^, suggest that such MM differences can lead to statistically significantly different parameter estimates.

We furthermore find that under antiviral therapy with an integrase inhibitor (INI), the shape of the MM-predicted decay differs drastically between the assumption of a normally vs exponentially distributed duration of virus production. The true decays observed in HIV patients under INI therapy^6^ thus far can be reproduced under either assumptions (not shown here), albeit for different parameter values. As such, it is not currently possible to use this distinction to confirm the true duration of virus production by HIV-infected cells *in vivo*. However, under the assumption of a normally-distributed virus-production phase, and when that phase is less than ~11 h in duration, the MM-predicted viral load decay is very sharp, occurring over a few hours, with a drop proportional to the efficacy of the INI therapy. This pattern cannot be reproduced under the assumption of an exponential duration of virus-production. Observing such a pattern under INI therapy would require more frequent sampling early after therapy initiation, and would provide a strong confirmation that the duration of virus production by HIV-infected cells *in vivo* follow a normal-like distribution.

A normally distributed duration of virus production indicates that the time of cessation of virus production by SHIV-infected cells *in vitro* occurs some relatively fixed amount of time after the start of virus production and release. This implies that the cause of this cessation is likely a consequence of that production and release process, such as the accumulation of damage (e.g., cytotoxicity, host cell resource exhaustion) or signalling (e.g., down-regulation due to virus replication-triggered cytokines). Recently, measuring intracellular HIV-1 replication kinetics alongside gene expression, with high temporal resolution, has become possible^15^. Such methodology would enable investigation of the detailed apoptotic gene expression profile during HIV-1 infection. This, in turn, would permit accurate quantification of the duration of virus progeny production and the mechanisms behind its cessation.

Our results are based on analysis of *in vitro* infection of HSC-F cells with SHIV-KS661. However, in the context of an *in vivo* HIV infection, the host immune response, absent in our *in vitro* analysis, might alter the distribution and duration of the virus-production phase. For example, killing of virus-producing cells by the host immune response might lead to an exponentially distributed virus-producing phase, but only if HIV-infected cells which have just begun virus production are killed at the same rates as those which have been producing HIV progeny for a while. It would furthermore require that a majority of HIV-producing cells cease virus production because of that host immune response so that cells exhibiting an exponentially distributed infectious lifespan dominates over those exhibiting their ‘natural’, normal-like duration of virus production. Viral load decay rates — believed to be proportional to the duration of the virus production phase — were reported to be the same in the presence and absence of CD8^+^ T cells in macaques chronically infected with SIV under combination antiretroviral therapy^16^,^17^,^18^. This suggests that at least the killing of SIV-infected cells by CD8^+^ T cell *in vivo* would not significantly affect virus-production duration. Additionally, while the rate of host-mediated killing of HIV-infected cells is high in the acute phase of an HIV infection, this rate is likely much lower in the chronic phase of the disease, when antiviral therapy is administered and studied. Using a mathematical modelling analysis, Ganusov *et al*.^19^ found a large decrease in the rate of viral escape from cytotoxic T lymphocyte (CTL) killing between the acute and chronic phases of HIV-1 infections and suggest this would be consistent with a decrease in the magnitude of epitope-specific CTL responses. For all these reasons, it is not inconceivable that the duration of virus production by HIV-infected T cells *in vivo* also follows a normal distribution, like that characterized herein for the SHIV-infection of HSC-F cells *in vitro*.

## Methods

### Quantification of viable and infected cells

These experimental procedures were outlined in ref. 1, but are repeated here for completeness. Virus infection of the HSC-F cells was measured by FACS analysis using markers for surface CD4 and intracellular SIV Nef antigen expression. The number of total and viable cells were first determined using an automated blood cell counter (F-820; Sysmex, Kobe, Japan). Viable HSC-F cells (gated by forward- and side-scatter results) were examined by flow cytometry to measure the surface CD4 and intracellular SIV Nef antigen expression. Cells were permeabilized with detergent-containing buffer (Permeabilizing Solution 2, BD Biosciences, San Jose, CA). The permeabilized cells were stained with phycoerythrin conjugated anti-human CD4 monoclonal antibody (Clone Nu-TH/I; Nichirei, Tokyo, Japan) and anti-SIV Nef monoclonal antibody (04-001, Santa Cruz Biotechnology, Santa Cruz, CA) labelled by Zenon Alexa Fluor 488 (Invitrogen, Carlsbad, CA), and analyzed on FACSCalibur (BD Biosciences, San Jose, CA).

### Quantification of viral load

These experimental procedures were outlined in ref. 1, but are repeated here for completeness. The total viral load was measured via real-time PCR quantification, as described previously^9^,^20^. Briefly, total RNA was isolated from the 100 fold diluted culture supernatants (140 μL) of virus-infected HSC-F cells with a QIAamp Viral RNA Mini kit (QIAGEN, Hilden, Germany). RT reactions and PCR were performed by a QuantiTect probe RT-PCR Kit (QIAGEN, Hilden, Germany) using the following primers for the gag region; SIV2-696F (5′-GGA AAT TAC CCA GTA CAA CAA ATAGG-3′) and SIV2-784R (5′-TCT ATC AAT TTT ACC CAGGCA TTT A-3′). A labelled probe, SIV2-731T (5′-Fam-TGTCCA CCT GCC ATT AAG CCC G-Tamra-3′), was used for detection of the PCR products. These reactions were performed with a Prism 7500 Sequence Detector (Applied Biosystems, Foster City, CA) and analyzed using the manufacturer’s software. For each run, a standard curve was generated from dilutions whose copy numbers were known, and the RNA in the culture supernatant samples was quantified based on the standard curve.

### Mathematical model (MM)

In order to compare distributions ranging from exponential, to normal-like, to Dirac delta-like for the duration spent by cells in the eclipse phase (*E*) and in the subsequent virus-producing infectious phase (*I*), we follow previous work^1^,^2^,^3^ and adopt a set of ordinary differential equations which make use of an Erlang distribution to represent these phases, namely


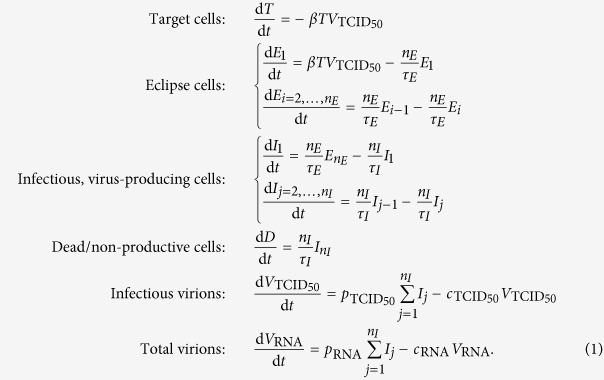


Herein, susceptible, target HSC-F cells are infected at rate *β* by infectious virus, 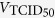, and upon successful infection, they enter the eclipse phase, 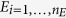 wherein they are SHIV-infected, but are not yet producing infectious virus. After an average time *τ*_*E*_ has elapsed since becoming infected, cells in the eclipse phase become infectious, 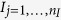, wherein they are assumed to produce infection (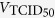) and total (*V*_RNA_) virus at a constant rates 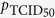 and *p*_RNA_, respectively. Ultimately, virus-producing infectious cells cease virus production, possibly undergoing apoptosis, after spending an average time *τ*_*I*_ in the virus-producing state. Total virus, *V*_RNA_, quantified via real-time quantitative PCR in units of vRNA/mL, was measured experimentally over the course of the infection. Infectious virus, 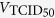, typically quantified via tissue culture infectious dose in units of TCID_50_/mL was not measured experimentally. The rate of cell infection is dependent on the concentration of infectious virus, not total virus. The ratio of infectious to total virus is not constant over the course of an *in vitro* SHIV infection due to the differing rates at which infectious virus loses infectivity compared to the rate at which total virus degrades or breaks down, i.e., 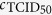 for 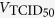 and *c*_RNA_ for *V*_RNA_ where 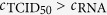. Therefore, in MM (1), both the total and infectious virus population is accounted for: the former for comparison to experimental measurements, the latter to appropriately capture infection kinetics.

The time spent by newly infected cells in each of the eclipse and virus-producing phases is represented in MM (1) by the Erlang distribution, a special case of the Gamma distribution in which the shape parameter (here *n*_*E*_ and *n*_*I*_) can only take on integer values. In Eqn. (1), the eclipse (or infectious) phase is separated into *n*_*E*_ (or *n*_*I*_) separate compartments, each lasting an exponentially-distributed time of equal average length *τ*_*E*_/*n*_*E*_ (or *τ*_*I*_/*n*_*I*_). As such, cells will spend on average *τ*_*E*_ (or *τ*_*I*_) time in the eclipse (or infectious) state prior to transitioning to the infectious (or dead) state.

We also define the following, additional, derived parameters which are computed from the base MM parameters introduced above. The infecting time, which corresponds to the time elapsed between the production of the first virion by an infected cell and the infection of the first cell by that progeny^2^, is computed as


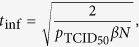


where *N* = 10^6^ is the number of cells in the culture. The basic reproductive number, which corresponds to the number of secondary infections resulting from a single infected cell over its virus-producing lifespan in a fully susceptible cell population is computed as


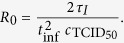


### Experimental and mathematically simulated *in vitro* SHIV infections

Experimentally, HSC-F cells were incubated with SHIV-KS661 and centrifuged at 4,000 rpm for 1 h at 25 °C, then rinsed three times as described in ref. 1. In order to computationally reproduce the experimental infection of HSC-F cells with SHIV-KS661, we used MM (1) with the following initial conditions. We consider the rinse time to correspond to *t* = 0 such that the infection, and therefore the mathematical simulation, begins at time *t* = −1 h, followed by a rinse at time *t* = 0, after which the infection is allowed to proceed without further intervention. This is because each sample (time point measurements of virus or cell viability or cell infection status) are taken from separate wells (independent replicates) such that each time point corresponds to an infection left undisturbed since rinsing, up until sampling. Thus, at time *t* = −1 h, we assume that no cell is initially infected (*E*_*i*_ = *I*_*j*_ = *D* = 0). We further assume that not all HSC-F cells are susceptible to SHIV infection (*T*(−1 h) = 0.9 × 10^6^), because ~10% of cells appear to have remained uninfected by the end of the experiment (see Fig. 2). We found that this assumption (90% rather than 100% cell susceptibility to infection) was better supported (significantly better SSR) by the data.

The experimental infections were preformed at five different virus concentrations, namely at an undiluted infectious SHIV-KS661 concentration of 4.2 TCID_50_/cell (Base MOI), and at serial 2-fold dilutions down to 2.1, 1.1, 0.53, and 0.26 TCID_50_/cell in four additional experiments. Mathematically, this is represented by setting the initial infectious virus concentration to 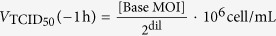, where dil = 0, 1, 2, 3, 4 is the dilution factor of the initial inoculum which is used undiluted (dil = 0), or diluted by 2-fold in four additional experiments. As such, our Base MOI should correspond to an initial virus inoculum of 4.2 × 10^6^ TCID_50_/mL. However, since there is poor correspondence between virus infectiousness across separate experiments^3^, i.e. TCID_50_ is a relative rather than an absolute measure of virus infectiousness^21^,^22^, the true Base MOI was left as a free parameter to be determined. The initial total virus concentration was set to 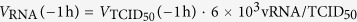 based on the ratio of 6,000 vRNA per TCID_50_ in our SHIV-KS661 samples, specifically, the ratio of the initial (*t* = 0) data points in Fig. 2 of Iwami *et al*.^9^.

After the 1 h incubation period, the cells are rinsed three times to remove excess virus. Based on the experimental measurements presented in Fig. 2, it would appear that at high virus inocula (MOI = 4.2, and 2.1 TCID_50_/cell), the residual virus post-rinse was not proportional to the inoculum, but rather seemed to reduce to the residual virus down to ~1.4 × 10^9^ vRNA/mL, whereas at the lower virus inocula, the rinse did seem to result in residual virus proportional to the inocula. Therefore, in our MM, we simulate the rinse by multiplying both 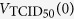 and *V*_RNA_(0) at time *t* = 0 by 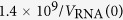 which is proportional to the true rinse. Since only total virus is measured (*V*_RNA_), yet has no impact on our simulated infection kinetics which instead depends on infectious virus 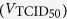, application of a proportional rather than an absolute rinsing factor merely means that the units of the rates of infectious virus production 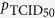 and infectivity *β* will be relatively, rather than absolutely accurate, which is always the case^21^.

In addition to its initial conditions, MM (1) contains a total of 9 parameters: *β*, 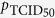, *p*_RNA_, 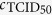, *c*_RNA_, *τ*_*E*_, *τ*_*I*_, *n*_*E*_, and *n*_*I*_. From our previous work reported in Iwami *et al*.^9^, we have determined the rate of loss of infectious and total SHIV-KS661 virions to be 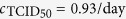 and 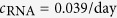, respectively, and the rate of infectious virus production to be 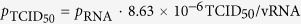, which we use since infectious virus is not explicitly measured in the experiments analyzed herein. This leaves a total of 6 parameters (*β, p*_RNA_, *τ*_*E*_, *τ*_*I*_, *n*_*E*_, *n*_*I*_) and one initial condition (Base MOI) to be estimated by our analysis. In performing the nonlinear least-square fits (Figs 2 and 3), we fixed *n*_*E*_ and *n*_*I*_ to various integer values ∈ [1, 100], while allowing the remaining 5 quantities to vary. The best-fit parameters obtained for the exponential (*n*_*I*_ = 1) and best fit for a normal-like distribution (*n*_*I*_ = 20) are presented in Table 2. In constructing the Markov chain Monte Carlo (Fig. 3), we allowed all 7 quantities to vary. The parameters obtained through the MCMC process are presented in Table 1.

In matching the experimental data to variables of our MMs, we compared the experimentally quantified total virus concentration via real-time quantitative PCR (vRNA/mL) to MM variable *V*_RNA_, compared the fraction of virus-producing cells tagged via anti-SIV Nef monoclonal antibodies and quantified via FACS to the sum of all MM variables corresponding to the fraction of cells in the infectious, virus-producing phase 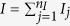, and compared the fraction of non-viable cells quantified using FACS gated by forward- and side-scatter to MM variable *D* corresponding to the fraction of dead cells. In our nonlinear least-square regressions to identify the best-fit, 6-parameter set for two different values of *n*_*I*_, we aimed to maximize the goodness-of-fit by minimizing the sum-of-squared residuals (SSR) between the experimental data, weighted for each of *V*_RNA_, *I*, and *D* as follows:


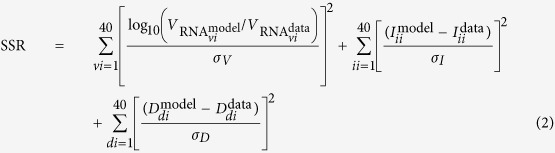


where 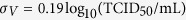, *σ*_*I*_ = 0.047, and *σ*_*D*_ = 0.080 correspond to the standard deviations of the pooled residuals for each of 
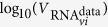
, 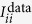 and 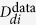, respectively, for 100 best fits performed for *n*_*I*_ ∈ [1, 100]. *I*_*ii*_ and *D*_*di*_ in the above correspond to fraction rather than number of cells. The SSR calculation directly uses the fraction of cells, and no transformation (e.g. logit function) is applied in calculating the SSR for the fractions of infectious or dead cells. This is because we found that the distribution of the best-fit residuals of the cell fraction data resembles a Gaussian while that for the residuals of the logit-transformed cell fraction data does not (not shown).

The nonlinear regression was performed using successive applications of python’s scipy implementations of the Levenberg-Marquardt (scipy.optimize.leastsq) and Nelder-Mead downhill simplex (scipy.optimize.fmin) methods. Additionally, we used emcee, a python module implementation of the Markov chain Monte Carlo (MCMC) method^23^, to identify the posterior distributions for all seven MM parameters from a total of >7,000,000 MCMC-accepted parameter sets. In constructing the MCMC chains, exp(−SSR/2) was used as the likelihood function for each set of parameters (dimensions), where the SSR is computed as per Eqn. (2). The ‘Best-fit likelihood’ (BFL) graph which appears in Fig. 3(k) was computed as exp(−SSR/2) of the minimum SSR obtained via a best-fit for each individual value of *n*_*I*_ ∈ [1, 100], divided by the likelihood of the very best fit (*n*_*I*_ = 1) so that the latter’s likelihood is one.

### Simulation of *in vivo* antiviral therapies with various modes of action

To simulate therapy with an integrase inhibitor (*ε*_IN_), we modified MM (1) as follows


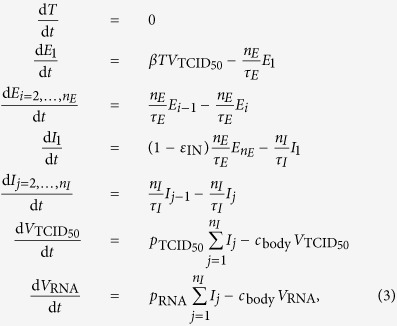


where 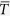 is the steady-state level of CD4^+^ T cells in patients chronically infected with HIV, i.e. we set 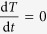 and simulate only the early viral decay post therapy initiation. The new parameter 
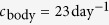
 is the rate of clearance of HIV from the plasma of chronically infected HIV patients, as previously estimated^7^. Because 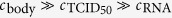, the rates of loss of infectivity 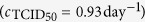 and viral degradation 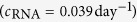 can be safely neglected.

The steady-state of MM (3) prior to therapy initiation is given by


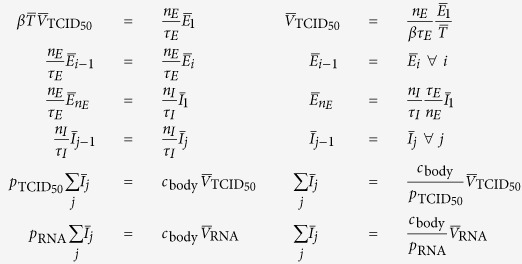


where we set *τ*_*E*_ = 19 h, *τ*_*I*_ = 11 h, *n*_*E*_ = 1 or 4 (exponential or fat-tailed eclipse phase), and *n*_*I*_ = 1 or 90 (exponentially vs normally distributed virus-producing lifespan), based on Table 1. Additionally, we set 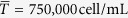 and 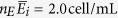 based on refs 24,25. We use 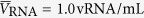 since the value of this variable is not important to the MM-predicted kinetics and is equivalent to rescaling the viral load to its starting, steady state value. As in MM (1), we set 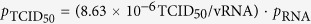. Following the steady-state equations above, we have


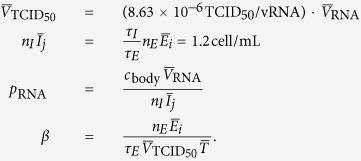


## Additional Information

**How to cite this article:** Beauchemin, C. A. A. *et al*. Duration of SHIV production by infected cells is not exponentially distributed: Implications for estimates of infection parameters and antiviral efficacy. *Sci. Rep.* **7**, 42765; doi: 10.1038/srep42765 (2017).

**Publisher's note:** Springer Nature remains neutral with regard to jurisdictional claims in published maps and institutional affiliations.

## Supplementary Material

Supplementary Materials

## Figures and Tables

**Table 1:** Parameters characterizing HSC-F cell infection by SHIV-KS661.

Parameter	Value [95% CR]^a^
Eclipse phase, *τ*_*E*_ (h)	19 [18, 20]
Virus-production (infectious) phase, *τ*_*I*_ (h)	11 [9.8, 12]
Infected lifespan, *τ*_*E*_ + *τ*_*I*_ (h)	30 [28, 31]
Prod. rate, *p*_RNA_ 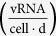	10^3.7^ ^[3.6, 3.8]^
Infectiousness, *β* 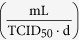	10^−4.1^ ^[−4.3, −3.9]^
Base MOI 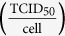	0.41 [0.30, 0.58]
Infecting time, *t*_inf_ (h)	18 [14, 25]
Basic repro. num., *R*_0_	1.7 [0.91, 2.9]
Burst size, *p*_RNA_ · *τ*_*I*_ 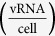	10^3.4^ [3.2, 3.5]
# eclipse eqns., *n*_*E*_	4 [3, 5]
# virus-production eqns., *n*_*I*_	12 [6, 97]

^a^Mode of the PLD and [Bayesian 95% credible region (CR)].

**Table 2:** Best-fit parameters for exponential vs normal-like virus production phase*.

Parameters	Virus-production phase distribution	
	Exponential	Normal-like
Eclipse phase, *τ*_*E*_ (h)	17.1	19.0
Infectious, virus-production phase, *τ*_*I*_ (h)	14.0	11.0
Infected lifespan, *τ*_*E*_ + *τ*_*I*_ (h)	31.0	30.0
Prod. rate, *p*_RNA_ 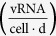	10^3.67^	10^3.69^
Infectiousness, *β* 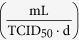	10^−4.12^	10^−4.11^
Base MOI 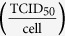	0.429	0.410
Infecting time, *t*_inf_ (h)	19.4	18.7
Basic repro. num., *R*_0_	1.91	1.62
Burst size, *p*_RNA_ · *τ*_*I*_ 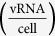	10^3.44^	10^3.35^
# eclipse eqns, *n*_*E*_	5	4
# virus-production eqns, *n*_*I*_ [FIXED]	1	20
Goodness of fit (SSR)	135	115

^*^Best fits are shown in Fig. 2.
