# Supplementary material for: Duration of SHIV production by infected cells is not exponentially distributed: Implications for estimates of infection parameters and antiviral efficacy
Source: Sci Rep. 2017 Feb 16;7:42765. doi: 10.1038/srep42765 (PMC5311941; doi:10.1038/srep42765)
Supplement: Supplementary Materials [file srep42765-s1.pdf]

Supplementary Material for:

Duration of SHIV production by infected cells is not exponentially distributed: Implications for estimates of infection parameters and antiviral efficacy

Catherine A. A. Beauchemin[1,2,*], Tomoyuki Miura[3], and Shingo Iwami[4,5]

1 Department of Physics, Ryerson University, Toronto, M5B 2K3, Canada
2 Interdisciplinary Theoretical Science (iTHES) Research Group, RIKEN, Wako, 351-0198, Japan
3 Institute for Virus Research, Kyoto University, Kyoto, 606-8507, Japan
4 Department of Biology, Kyushu University, Fukuoka, 819-0395, Japan
3 CREST and PRESTO, Japan Science and Technology Agency (JST), Saitama, 332-0012, Japan
***** Corresponding author. Email: `cbeau@ryerson.ca`

January 5, 2017

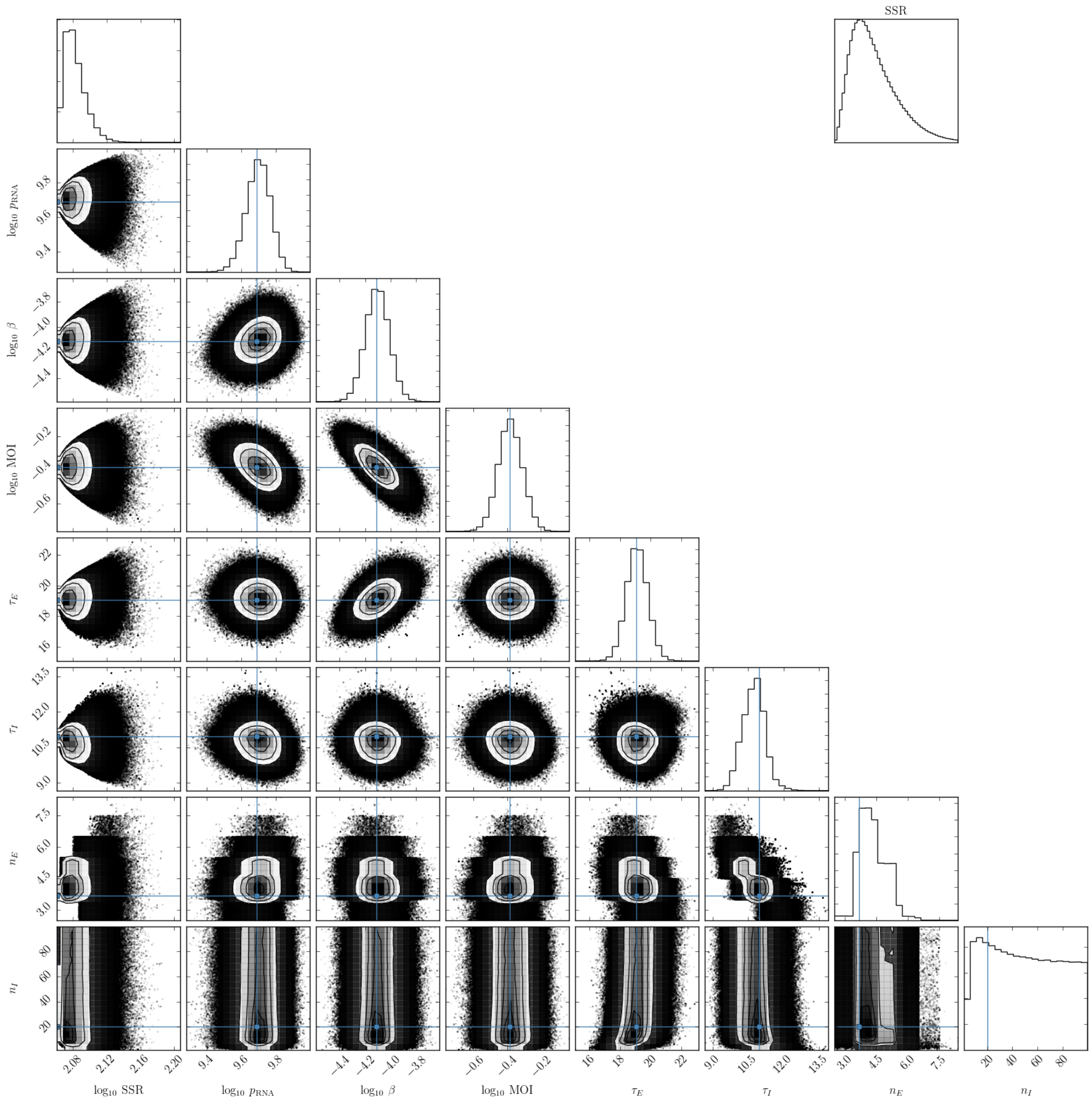

Figure S1: **Two-parameter posterior likelihood distributions**. The two-parameter posterior likelihood distributions (PLDs) for each of the $> 7,000,000$ MCMC-accepted parameter sets are shown for each pair of MCMC-determined parameters and their associated sum of squared residuals (SSR). The symbols are as follows: virus production rate (p_{RNA}), virus infectivity (β), base multiplicity of infection (MOI), duration of the eclipse (τ_E) and virus-producing (τ_I) phases, and number of equations for the eclipse (n_E) and virus-producing (n_I) phases. Vertical or horizontal distributions indicate the absence of correlations. Diagonal distributions indicate correlations and possible lack of identifiability of some parameters. Generally, the correlations are minor and the PLDs are narrow indicating good identifiability. The larger correlations are those between (MOI,β) and (n_E,τ_I). Milder correlations are present between (MOI,p_{RNA}), (τ_E,β), (n_E,τ_E) and between (n_I,τ_E) and (n_I,τ_I) when $n_I < 10$ or so.
